# One Room Schoolhouse: A Novel Intervention for Inspired Academic Half-Day Learning in Distributed Campus Settings

**DOI:** 10.1177/23821205211029462

**Published:** 2021-07-08

**Authors:** Sheila Harms, Anita Acai, Bryce JM Bogie, Meghan M McConnell, Ben McCutchen, Robyn Fallen, JoAnn Corey, Natasha Snelgrove

**Affiliations:** 1 Department of Psychiatry and Behavioural Neurosciences, McMaster University, Hamilton, ON, Canada; 2 Faculty of Medicine & The Royal’s Institute of Mental Health Research, University of Ottawa, Ottawa, ON, Canada; 3 Department of Innovation in Medical Education, University of Ottawa, Ottawa, ON, Canada; 4 Department of Anesthesiology and Pain Medicine, University of Ottawa, Ottawa, ON, Canada

**Keywords:** Academic half days, engagement, learning, distributed campus, residency education

## Abstract

**Introduction:**

Some studies on academic half days (AHDs) suggest that learning in this context is associated with a lack of educational engagement. This challenge may be amplified in distributed campus settings, where geographical disadvantages demand reliance on videoconferencing or considerable time spent travelling to in-person learning events. Concerns about the educational effectiveness of AHDs by learners within our distributed campus setting led to the development and evaluation of the One Room Schoolhouse (ORS), a unique, evidence-informed, community-based curriculum that partially replaced the AHD sessions delivered at the main campus. It was hypothesized that creating an AHD experience that was clinically reflective of the community in which residents practiced and where residents were given the autonomy to implement novel pedagogical elements would result in better test scores and improved learner satisfaction among ORS learners.

**Methods:**

The ORS was implemented at McMaster University’s Waterloo Regional Campus in 2017. Residents across training cohorts (N = 9) engaged in co-learning based on scenarios co-developed from clinical experiences within the region. The learning approach relied on multiple, evidence-informed pedagogical strategies. A multi-method approach was used to evaluate the ORS curriculum. Between-subject analyses of variance were used to compare scores on practice exams (COPE and PRITE), in-training assessment reports (ITARs), and objective structured clinical exams (OSCEs) between learners who took part in the ORS and learners at the main campus. A semi-structured focus group probing residents’ experiences with the ORS was analyzed using interpretive description.

**Results:**

ORS learners significantly outperformed learners at the main campus on the November OSCE (*p* = .02), but not on the COPE, PRITE, ITARs, or September OSCE (*p*’s < .05). Qualitative themes suggested advantages of the ORS in inspiring learning, engaging learners, and improving self-confidence in knowledge acquisition. These findings are aligned with the broader literature on learner agency, social development, and communities of practice.

**Conclusion:**

While the quantitative data only showed a significant difference between the 2 curricula on 1 measure (ie, the November OSCE), the qualitative findings offered an opportunity for educators to reimagine what medical education might consist of beyond the confines of a “traditional” AHD. Creating opportunities to enhance personal agency when acquiring knowledge, inspiring engagement about patient-related problems, and incorporating interdisciplinary learning through community engagement were critical pedagogical elements that were attributed to the success of the ORS.

## Introduction

A standardized definition of academic half days (AHDs) has not been agreed upon in medical education literature. However, one of the few definitions that is available defines AHDs as regularly-scheduled teaching events that are directed at residents and offer protected time away from patient care to deliver key content in residency training.^1^ In some contexts, AHDs were reportedly introduced to replace noon-hour teaching sessions, which were associated with a number of challenges including poor resident attendance, clinical interruptions, time constraints to deliver content, and ineffective pedagogical strategies.^2^ Replacing noon-hour teaching sessions with AHDs can help resolve some of the challenges around clinical interruptions as AHDs offer protected time for resident learning^3,4^; however, this approach may not fully resolve issues around a lack of educational engagement. In some settings, ongoing challenges with learning satisfaction and effectiveness continue to be associated with AHD delivery.^1^ For example, a study of Canadian resident perceptions of AHDs found that engagement during these sessions was often poor, especially when passive instructional methods were used or when residents were not fully integrated into the learning experience, such as when videoconferencing into the sessions from distributed sites.^5^ Residents in this same study indicated that content delivered during AHDs was rarely retained.^5^ Moreover, while AHDs were designed to offer “protected time” for resident learning, this did not always occur as some residents felt a responsibility to prioritize their clinical duties over classroom-based learning, while other residents used the time to run personal errands.^5^ Similar concerns have been reported in other studies,^1,6^ with 1 study also identifying program-specific differences in the pedagogical strategies used during AHDs, which translated to differences in perceived relevance among residents.^6^

In general, AHD curricula and studies on this topic have largely been atheoretical in nature, with descriptions of AHD activities and interventions only being superficially linked to pedagogical literature. Some residency programs have attempted to mitigate the challenges of promoting learning during AHDs by building in a range of pedagogies that emphasize active engagement in the learning process, including increased interactions with teachers and peers to foster social cohesion and professional identity formation.^5,7^ Examples include case-based learning, resident-led teaching sessions, and simulation exercises.^8-10^ Active learning strategies have gained wide support in the medical education literature, including the “flipped classroom” model. This format is a commonly used approach in which knowledge acquisition and preparation are performed in advance, on learners’ own time, so that the focus of classroom-based teaching can be focused on problem solving and application-based skills.^10^ This allows for didactic lectures to be replaced by more active pedagogies, such as those mentioned earlier.^11-13^ For example, a recent meta-analysis by Hew and Lo^11^ revealed that the flipped classroom approach produced better learning outcomes than didactic lectures and is preferred by most learners. Some studies have even suggested links between case-based learning and improved patient care.^14-16^ Critically, case-based learning differs from other, similar learning approaches, such as problem-based learning, because it is more structured through the use of specific learning objectives and greater teacher involvement.^17^ As such, it may be more effective for experienced learners with existing foundational knowledge of the material.^7^

The AHDs in the distributed campus setting of the Psychiatry Postgraduate Training Program at McMaster University’s Waterloo Regional Campus (WRC) struggled with many of the same challenges described earlier. Although the delivery of AHD sessions at the main campus has attempted to use pedagogically robust strategies, the delivery of the AHD sessions has largely been varied, depending on facilitator skills and preferences. Additionally, the program’s original AHD model relied on distributed campus residents to either drive 60 minutes or video conference into the main campus site to participate in AHDs, which detracted from their learning experience. In response to these challenges, the program leadership opted to replace half of the WRC residents’ AHD sessions with a unique, community-based curriculum referred to as the One Room Schoolhouse (ORS), with the remaining half being delivered through video conference with the option to travel to in-person sessions. The curriculum was intentionally designed using best practices drawn from the pedagogical literature to fill a gap in the existing scholarship on AHDs. Moreover, the curriculum was intended to address concerns about participating in AHD learning through videoconference by capitalizing on the educational resources inherent in the skills of postgraduate learners in a distributed campus setting, and by highlighting learning opportunities within the unique community context of the distributed campus. The purpose of the current study was to assess the effectiveness of the ORS as it related to learner performance using different components of a programmatic assessment strategy, as well as a qualitative focus group with learners who engaged in the ORS.

## Methods

### Context

McMaster University is a mid-sized academic health sciences center located in Hamilton, Ontario, Canada. At the time of the study, the residency program in psychiatry consisted of 44 residents distributed across 1 main campus in Hamilton (N = 35) and 1 smaller, distributed campus in a neighboring community, known as the WRC (N = 9). The ORS was implemented at the WRC in 2017, while the Hamilton Campus continued to operate under its standard AHD model.

### ORS curriculum

The creation of the ORS curriculum reflected a radical shift in the delivery of academic teaching sessions in the Psychiatry Postgraduate Training Program. The learning approach relied on multiple, evidence-informed pedagogical strategies. All WRC residents agreed to participate in the ORS and formed a small, fixed group consisting of learners from all levels of training (ie, postgraduate years [PGYs] 2 to 5) and relied on the ORS to replace half of the AHDs delivered at the main campus. The amount of time spent in AHD learning was equal between the 2 campuses. ORS residents were also able to join AHDs at the main campus if the timing did not conflict with ORS participation; however, very few ORS residents chose to do so. ORS residents worked collaboratively with faculty to write up complex clinical scenarios encountered by learners in the community using a case-based approach. Each module consisted of 2 to 3 weekly academic sessions relevant to a specific complex case or problem. Key learning objectives were provided to orient learners to expectations set forth by the Royal College of Physicians and Surgeons of Canada (RCPSC). However, residents ultimately set their own personal and group learning objectives, which were then researched independently between sessions to achieve the knowledge, skills, and attitudes relevant to learning objectives. In this way, the varying learning needs of residents at different stages of training could be addressed and met.

The first ORS module occurred in November 2017 and there were 3 ORS modules delivered each academic year, which replaced half of the AHD curriculum delivered at the main campus. During the first session of each module, residents engaged in objective setting. Residents used the second session to engage in face-to-face co-learning and teaching with faculty facilitation. The third session was used to complete any remaining learning objectives, and to practice specific skills applicable to the clinical problems and settings. Unique pedagogical opportunities included site visits to relevant community agencies, such as homeless shelters, as well as meetings with street outreach staff, family caregivers of individuals with neurocognitive disorders, and other community leaders with the intent of learning from others not traditionally associated with psychiatric education. Psychiatry residents were encouraged to learn about the role and contributions of psychiatry in the community, with a particular focus on treating individuals with severe and persistent mental illness to better understand the subsequent impact of mental illness on the person, the family, and the community.

The completion of all other scholarly training requirements offered through the core curriculum delivered at the main campus (ie, diagnostic interviewing, psychiatric formulation, practicing of objective structured clinical examination [OSCE] skills, evidence-based medicine) was designed to be anchored to the ORS modules, thereby reinforcing concepts within a singular case as well as offering opportunities to capitalize on repetition and consolidation of ideas. For example, the OSCE practice cases were designed with reference to topics within the current ORS session. The group learning opportunities were intended to be linked to community clinical encounters to reduce the fragmentation that often occurs in postgraduate medical education. At the main campus, all additional scholarly training requirements (ie, OSCE practice, diagnostic interviewing, etc.) were offered and delivered as part of the standard AHD experience, but were not thematically tied to a particular clinical case.

### Evaluation

Evaluation of the ORS consisted of 2 parts: a quantitative analysis of resident examination and clinical performance as well as a qualitative analysis of resident perceptions via a semi-structured focus group. The study did not formally use a mixed-methods approach as data collection was not initially conceptualized in an integrated manner^18^; instead, existing quantitative data were later supplemented by a focus group. The authors adopted an epistemological orientation where social and constructivist learning theories, which suggest that knowledge is subjective and there is not a singular “truth” to be discovered, were relied upon to develop the ORS and to interpret data.^19^

#### Quantitative measures: Examination and clinical performance

The Coordinators of Psychiatric Education (COPE) Examination and the Psychiatry Resident-In-Training Examination (PRITE) are both standardized, multiple-choice examinations taken by psychiatric trainees at McMaster University once per year to help them prepare for their national licensing examination. Both are national examinations; the COPE Examination is Canadian while the PRITE is American. In addition, the program offers 2 OSCEs per year, once in the spring and once in the fall, to assess trainee competence on core skills through their second to fifth years of training. Both OSCEs are also used as preparatory tools for the national licensing examination administered through the RCPSC.

Dependent measures included resident scores on both written examinations and the mock OSCEs, as well as their scores on in-training assessment reports (ITARs). These scores were extracted, de-identified, and used to compare performance between the WRC, where the ORS curriculum was implemented, and the Hamilton Campus, which continued to operate using its standard AHD model. Where available, data from each quantitative measure were collected for each resident during the 2016, 2017, and 2018 academic years. Separate, 2-way, between-group analyses of variance (ANOVAs) were conducted for each dependent measure, with postgraduate year (PGY: 2, 3, 4, or 5) and campus (WRC or Hamilton) as the independent measures. Statistical analyses were conducted using IBM’s Statistical Package for Social Sciences (SPSS®). Statistical significance was considered at *p* < .05.

#### Qualitative examination of resident perceptions

A semi-structured focus group with WRC residents was conducted in July 2019 to further explore resident perceptions of the ORS curriculum. A focus group was selected over individual interviews to allow residents to share and build upon 1 another’s perspectives.^20^ A semi-structured interview guide was developed by 2 researchers (BB and SH) to explore residents’ perceptions of, and experiences with, the ORS in greater detail (see Supplemental Material 1). All residents who were a part of the ORS curriculum were sent an email invitation to participate in the focus group. Participation was entirely voluntary, with no impact on residents’ standing in the residency program. The focus group was moderated by BB, a researcher external to the residency training program. The resulting audio recording was transcribed by a professional transcriptionist who was also external to the program.

Analysis of the de-identified focus group transcript employed interpretive description^21^ to generate codes and themes. Interpretive description allows researchers to meaningfully explore participants’ experiences, including both individual and group perspectives.^21^ Given its focus on experience, this approach is also conducive to generating practical applications that can help advance health professions education.^21^ Two researchers (AA, a PhD-trained education scientist, and SH, an experienced qualitative researcher and clinical educator) independently coded the focus group transcript using an inductive approach and met to co-develop themes. Reflexivity through open dialogue and reflection, independent scrutiny through the involvement of multiple experts as part of the analytic and authorship team, and the creation of an audit trail helped ensure rigor throughout the analytic process.^21^

### Ethics approval

This study received approval from the Hamilton Integrated Research Ethics Board (HiREB-5159).

## Results

### Quantitative measures: Examination and clinical performance

Descriptive statistics for each dependent measure, including the number of data points (ie, n) available for each, are presented in Table 1. Results of the 2-way, between-group ANOVA showed that residents engaged in the ORS curriculum did not perform significantly differently than those at the main campus on the COPE, *F*(1,94) = 2.70, *p* = .10, nor on the PRITE examinations, *F*(1,91) = 1.50, *p* = .23. Findings for the PRITE remained consistent even after applying *z*-transformations to the data to control for variations in test difficulty across academic years. A separate, 2-way, between-group ANOVA showed that residents engaged in the ORS curriculum also did not perform significantly differently than those at the main campus on their ITARs, *F*(1,193) = .01, *p* = .92, nor on the April OSCE, *F*(1,84) = 1.60, *p* = .21. However, there was a significant effect of curriculum type on the November OSCE, such that residents engaged in the ORS curriculum performed significantly better than those at the main campus, *F*(1,87) = 6.10, *p* = .02.

**Table 1.** Descriptive statistics for practice exams (COPE and PRITE), ITARs, and OSCEs for residents at the Waterloo and Hamilton campuses, 2016 to 2018. Data are presented as mean (SD), with n representing the number of data points per PGY level within each campus.

Assessment	Campus			
	Waterloo	n	Hamilton	n
COPE^a^				
PGY2	67.0 (4.7)	6	63.8 (6.2)	16
PGY3	65.7 (7.5)	7	68.4 (6.0)	19
PGY4	72.1 (3.6)	7	68.5 (5.9)	20
PGY5	81.8 (2.1)	4	75.8 (8.2)	23
PRITE^a^				
PGY2	61.1 (5.8)	6	58.1 (5.3)	21
PGY3	63.2 (1.1)	5	61.3 (5.0)	21
PGY4	62.2 (4.5)	5	62.7 (6.2)	21
PGY5	64.8 (6.4)	4	62.4 (5.6)	16
ITARs^b^				
PGY2	4.2 (0.5)	17	4.4 (0.5)	88
PGY3	4.3 (0.5)	14	4.2 (0.6)	58
PGY4	4.6 (0.3)	12	4.4 (0.7)	17
PGY5	–	–	–	–
OSCE^a^, April				
PGY2	71.9 (6.7)	3	70.0 (8.7)	17
PGY3	77.3 (9.2)	4	74.4 (5.6)	18
PGY4	80.0 (4.0)	4	76.5 (9.2)	21
PGY5	88.5 (4.9)	3	85.2 (7.5)	22
OSCE^a^, November				
PGY2	65.2 (4.9)	5	64.9 (6.7)	20
PGY3	83.0 (8.4)	4	73.8 (8.6)	19
PGY4	81.1 (5.7)	5	76.8 (5.7)	19
PGY5	83.5 (4.2)	6	80.1 (7.3)	17

a Assessment score is represented as a total percentage.

b Assessment score is represented as the average score across all 7 CanMEDS roles (each individual role was scored out of 5 points).

### Qualitative findings: Resident perceptions

The qualitative findings reflect a perspective from most residents who participated in the ORS (ie, 5 of 9 residents). Analysis of the focus group data resulted in 6 themes, which are discussed below. These themes can broadly be thought of in 2 overarching headings that (1) speak to the transformative nature of the ORS learner experiences and (2) highlight next steps in further developing this approach.

#### Theme 1: Academic half day—What’s not working

Residents were unanimous in their shared experience of the previous AHD model, which primarily relied on videoconferencing into the main campus for the delivery of academic content. Using this platform was described as an “*exercise in frustration tolerance*.” Although these sessions were intended to be interactive, there were many barriers, such as poor internet connectivity, presenters who were less skilled in engaging remote learners, and the use of purely didactic teaching strategies, which rarely resulted in satisfactory or meaningful learning:

*“I was very consistently let down. . . . One in part because early on we were using [a videoconferencing system] and it would fail half the time. So, you actually weren’t connected. And two because oftentimes it ends up being an informal discussion with the psychiatrist on the other end, which, when you are on a computer, is really hard to follow. . . . And eventually you just kind of tune them out and you are just there to get the checkmark attendance.”*

Learners were also cognizant of factors that negatively impacted the learning experience at the main campus, such as a focus on community resources that were geographically proximal to the main site but not the distributed site, or the coverage of “*esoteric*” lecture topics that appealed to only a small number of learners. Lastly, being able to articulate what was not working in the context of having a viable learning alternative to AHD sessions at the main campus was also described with a sense of relief and seen to promote learning independence.

#### Theme 2: ORS is a breath of fresh air—learning in action

Residents engaged in the ORS model of learning were consistently positive in their descriptions. Specifically, they noted that the principle of learning integration was key. This was experienced in many ways, including taking real-life cases and using them to expand knowledge and skills within their specific community context. This included seeking interdisciplinary perspectives and resources which was described as altering how empirically based facts and statistics were understood:

“*One of the things that is important is this level of integration. . . . Instead of . . . having a patient from a random unit to do diagnostic interviewing on, or to pick a random article and have it for evidence-based medicine, it was usually associated with whatever case we had reviewed. . . . [W]hen it is all wrapped up in a bow like that, [it] helps it integrate into more learning for longer.*”

Residents described a process of experiential learning that not only relied on their own clinical encounters as a fundamental starting point in developing cases, but also encouragement from their teachers to seek out additional experiences that would enhance their learning, such as conversations with community stakeholders. This approach was seen as a “breath of fresh air” that allowed them to supplement their understanding of specific psychiatric cases covered in the ORS with a deeper exploration of questions and issues through experiential learning. These pedagogical novelties that were not only permitted but encouraged in this model were described as a transformative alternative to AHD sessions at the main campus:

“. . . *it is kind of neat that each resident becomes a leader for the ORS and writes the case up and is able to be involved in the trajectory of the ORS, which I think also gives a completely different learning perspective . . . [and] is just something that you would never get [from AHD sessions at the main campus].*”

#### Theme 3: Learning as social engagement—within and beyond the classroom

Residents in the ORS were proud of being in a smaller community setting and articulated the community context as providing an important setting where relationships with community partners and stakeholders were important in meeting their patients’ needs. In this way, they described recognizing learning as a social experience that had not previously been accessible to them during AHD sessions at the main campus:

“. . . *we get to see each other and work as a group on these different things. And [we] have a social element of the curriculum, as well, that you miss in the formal curriculum in a distributed campus.*”

Residents described becoming more acutely aware of social accountability, recognizing that in their social interactions with community members, they are seen as leaders and need to be accountable to community expectations and needs, as well as to one another as professional colleagues. ORS participation also seemed to lead to greater cohesiveness among the residents themselves, acting as a bridge between the different levels of trainees and helping to “*cover any gaps that could potentially be missing*.” One resident made a comment about community engagement as an essential part of their learning, pointing to a realization that medical education goes far beyond the walls of hospital-based classrooms:

“*When you have applicability of the case towards . . . the community that you are working in, the hospitals that you are working in, and the staff that you are going to see throughout residency, it makes for much richer learning.”*

#### Theme 4: Tolerating uncertainty—leaving the expert behind

Residents described engaging in the ORS as an act of courage because it required them to miss out on what was being offered at the main campus. Although there was an acknowledgement that what was being offered at the main campus was generally ineffective in meeting their learning needs, a “fear of missing out” still registered for all the residents in choosing to participate in the ORS:

“*That could be the problem with a ‘choose-our-own’ curriculum because we . . . maybe aren’t aware of the rare thing that we don’t know enough about, and we don’t know enough to know that we need to know it.*”

In missing out on some of the lectures provided at the main campus, ORS learners were forced to reckon with the need to balance learning specific to the final credentialing examination based on RCPSC objectives and learning that was specific to the needs of the patient. Often, these 2 learning foci were in competition, requiring ORS participants to make a choice and subsequently experience a kind of uncertainty about how to focus their efforts:

“*I remember . . . when I was PGY2 and 3 and thinking, ‘Oh, I need this expert lecture; I need to attend the super pharm or the child or I need this invitation.’ And then I was PGY5 and it is not like I would have remembered any of those. So, I feel like the first four years of residency is better spent on more applicable things because you aren’t going to remember an expert lecture from PGY2.*”

ORS participants sometimes felt a responsibility to direct learning in a way that would benefit the patient represented in the clinical case. This tension represented a tautology in action. In other words, because the examination objectives were presumably written by experts, they represented expert knowledge required of the learners. However, this was sometimes at odds with the learner experience as it related to issues important to the patient and therefore required a gradual departure from “expert knowledge:”

“*My . . . perspective on residency is gradually you get to a point where you develop an expertise on all of these subjects and then you remember . . . that [expertise] is inherently flawed.*”

#### Theme 5: The learning self

All learners in the ORS spoke about gains made in their academic, personal, and professional lives as a result of being involved in the ORS. Specifically, they described having an improved sense of agency as it related to their learning but also with respect to taking ownership for patient problems. They also described being deeply engaged with learning, which paradoxically was surprising for them despite being in an academic environment. They noted that their self-confidence as a clinician who would be engaged in lifelong learning improved. ORS participants often felt as though they were on “the cusp” of learning a new concept or skill during ORS sessions. The supportive facilitators and the way that the cases were written allowed for a discovery of knowledge or skills previously unknown or out of reach to them:

“*It was nice to prove ourselves wrong over time, that we can have fulsome discussion about this case, and we can set our own objectives that are going to achieve our learning goals without having an expert saying, ‘This is what you need to learn.’ We were able to use our own clinical experiences to drive that.*”

In reflecting on their experiences with the ORS, participants noted that content is important but learning to learn was more important. In light of this, the increased learning workload associated with the ORS was determined by residents not only to be a positive encounter, but something that made their learning more rewarding. Participants also spoke very passionately about the need for ORS facilitation to be derived from local faculty for it to feel as though the project was uniquely situated entirely in the community context:

“*I know [we] have spoken about it a bunch of times, but my big shout out would be to [the facilitators]. I truly don’t think this curriculum would be what it is without them and their experience of being local residents—some of the first residents to go through this program and build some of the rotations here.*”

Lastly, residents spoke about the ability to compare ORS facilitators with AHD facilitators at the main campus and concluded that their experiences were of the highest caliber, further reinforcing the notion of value in the ORS experience.

#### Theme 6: Improving ORS—what’s next?

There were very few criticisms about the ORS or suggestions for improvement by the participants. Faculty development efforts were encouraged to train more facilitators to effectively engage with ORS modules. Participants also suggested that formal resident leadership roles for ORS be created in addition to having protected time to create and expand the curriculum.

## Discussion

This study assessed the effectiveness of the ORS as it related to learner performance using different components of a programmatic assessment strategy, as well as a qualitative focus group with learners who engaged in the ORS. Quantitative data only showed a significant difference between the 2 curricula on 1 measure: the November OSCE. However, the measures used to compare the ORS to AHD sessions at the main campus are blunt measuring tools and may benefit from additional information in the form of resident narratives to better understand the learner experience. Indeed, qualitative findings were consistently positive with respect to learner satisfaction, meaningfulness of learning encounters, and the degree of engagement and academic excitement about the learning that was occurring.

The overtly positive qualitative findings required an attempt at understanding why this phenomenon occurred. Learner satisfaction was a major theme, with a subtheme being learner agency and autonomy directed toward clinical encounters that were both familiar and shared across most of the participants. According to Bandura’s^22,23^ social learning theory, personal attributes interact with environmental contexts to influence behavior and degree of agency. Self-efficacy, learners’ “beliefs about their capabilities to produce levels of performance that exercise influence over events that affect their lives,”^24^ (p. 71) is an important part of agency. Thus, curricula that promote autonomy in learning and foster residents’ belief in themselves as legitimate sources of learning are foundational to developing agency. Being able to “curate” their own curriculum as part of the ORS allowed for a unique learning experience that likely enhanced learner satisfaction through encounters that mimicked the learning demands of practicing clinicians. In this way, ORS participants could move toward independent mastery in a specific patient encounter, reinforcing self-confidence, and mitigating the effects of uncertainty that are predictably experienced as part of learning medicine.

Vygotsky^25^ has also argued for the criticality of social interaction in learning through his idea of a “zone of proximal development.” This concept suggests that while students should be at the center of the learning experience, they may benefit from the support and guidance of a more experienced teacher as they progress toward independent mastery.^26^ ORS participants spoke about being able to tolerate the uncertainty related to a “fear of missing out” while their colleagues at the main campus were perceived to be doing work more strictly aligned with a RCPSC curriculum. In deciding to move forward with their learning objectives related to patient care, there was a forced opportunity to come to terms with the incomplete nature of knowledge. In other words, participants created their own encounter where they experientially arrived at the notion that they will never “know it all” and were forced to grapple with the anxiety of determining how much knowledge is enough knowledge in any given encounter. The social cohesion of the resident group coupled with supportive facilitators likely created an ability to collectively tolerate these uncertainties in a way that was both validating and reassuring. ORS participants also commented on how the social experience within the group deeply influenced the motivation to learn beyond the place where they would have historically stopped to “not let others down.” Vygotsky’s zone of proximal development suggests that learning is optimized when students’ experiences are supported by teachers (be they residents or faculty) who can offer guidance throughout the meaning-making process.^26,27^ This notion of “supported facilitation”^28^ was identified by participants as being central to the ORS curriculum.

Another aspect of the social learning that occurred in the ORS could be conceptualized as a community of practice, which is a more contemporary extension of social learning theory in which social engagement is used to support ongoing academic and professional development.^29^ In this theory, learning is inextricably tied with meaning and identity.^29^ As learners progress through the curriculum, they gradually develop their identities as physicians through a process of meaning-making that is linked to their broader social context.^29^ In the ORS, not only did learners have an opportunity to build a community with one another and with their facilitators, but the notion of community was extended to include others who may be central to the practice of psychiatry. These included interdisciplinary community partners, such as individuals from local shelters, law enforcement agencies, and geriatric support programs, who, through their involvement in the ORS curriculum, also became central actors in residents’ learning. This interdisciplinarity, combined with a breaking down of boundaries between different learner levels (ie, PGY) facilitated learning both within and beyond the walls of the classroom.

Another important finding was the role of geography in influencing the educational experience of participants in the ORS. Despite having access to the main campus teaching sessions through videoconferencing, there was an underlying sense of exclusion throughout AHDs taking place at the main campus. In other words, the physical distance from the main campus made them outsiders to learning, resulting in a sense of disengagement that was clearly described by the participants. There was a shared excitement about the idea of a learning community being built directly within the distributed campus, resulting in residents making concerted efforts to ensure success while also remaining mindful that they were part of a pilot program where there were expectations for quality. A respectful and friendly competition also emerged for the ORS group in comparing their learning outcomes with the main campus residents. This unintentional dynamic appeared to be helpful in motivating residents to ensure that their learning encounters were successful.

Overall, the data from this study help to orient educators in thinking about re-conceptualizing the AHD for postgraduate learners in medicine, whether it be through the implementation of a model like the ORS or the development of new curricula that rely on the same, underlying pedagogical principles. While the literature highlights numerous challenges associated with the delivery of quality AHD experiences, such as engagement^1^ and perceived relevance,^6^ the findings from the ORS suggest that there are pragmatic opportunities for potential improvement. One of the strengths of the ORS was intentional efforts to integrate the different kinds of learning opportunities (ie, diagnostic interviewing, psychiatric formulation, practicing of OSCE skills, evidence-based medicine) so that learning was topically linked and allowed for meaningful curricular alignment, which facilitated learning through repetition.^30^ Small group dynamics created a form of healthy competition among residents, a pedagogical strategy that was described as successful in enhancing motivation and accountability when group autonomy was explicitly built into the experience.^31^

While relying on case-based learning is not a novel concept, the notion of learners writing up complicated cases with the support of faculty proved to be an exercise that promoted learning before the actual small group learning occurred across iterative sessions. This raises the notion of efficiency in learning, whereby most, if not all, encounters can be explicitly made an opportunity for educational enhancement. Lastly, even as residency programs move toward competency-based medical education (CBME),^32^ there will continue to be a role for AHDs to teach learners content that would not otherwise be realistically encountered clinically. Thus, even in a CBME context, it will still be important to think about how to best use the time and effort required to make learning in an AHD context successful.

A limitation of this study included the fact that only WRC residents participated in the focus group, meaning that the experiences of learners engaged in the AHD curriculum at the main campus could not directly be compared. However, since WRC residents had engaged in AHD learning at the main campus prior to the implementation of the ORS, perspectives on this issue were gathered as part of the focus group reflected in this article. Furthermore, the quantitative data offered a numeric comparison of performance between the 2 groups, although these analyses may have been unavoidably underpowered due to the small number of learners at the WRC (N = 9). Nonetheless, it is encouraging that ORS residents outperformed residents at the main campus on at least 1 measure (ie, the November OSCE).

Another potential limitation is the Hawthorne effect.^33^ In other words, participants in the ORS were aware that they were engaging in a novel curriculum that was being studied, which may have prompted additional excitement and positivity during the focus group. It is also not possible to rule out the fact that the switch from videoconferencing to in-person learning prompted at least some of the positive effects of the ORS, as opposed to the other pedagogical elements described in this article. However, a mitigating factor for both potential limitations is that focus group participants gave detailed examples of why they found the ORS to be effective, many of which described specific pedagogical elements of the curriculum that went far beyond novelty and a shift away from videoconferencing.

Finally, the performance of learners at different levels (ie, PGY) within the ORS could not be compared due to the small number of learners in the ORS and the need to protect anonymity. As such, it was not possible to determine whether the curriculum worked equally well for residents at different levels of training. There is also the recognition that the ORS required significant resource allocation (eg, the development of cases, facilitators) and benefitted from a specific learning culture that may be more difficult to replicate at sites with a larger number of learners. Future research will investigate the feasibility of implementation in other residency contexts, including at the main campus, and whether any adaptations are needed to make the curriculum feasible for a larger number of learners.

## Conclusions

The purpose of this study was to address challenges with AHD delivery in a distributed campus setting where residents reported dissatisfaction and feeling disconnected from the learning sessions at the main campus. In response, a novel curriculum was developed based on the best available evidence. Evaluation data were compared between the main and distributed campus settings as a way of determining effectiveness of the newly developed AHD within the distributed campus setting. While the quantitative data only showed a difference between learners engaged in the ORS curriculum and learners at the main campus on 1 measure (ie, the November OSCE), qualitative findings suggested that a learning community had emerged at the distributed site, providing strong support for the value of the ORS beyond quantitative metrics. Specific themes related to learner satisfaction, the importance of building autonomy into learning, curating one’s own interdisciplinary curriculum, preparing learners for practice demands, tolerating the uncertainty of not knowing, as well as the role of social learning both within a group of learners and more broadly with community stakeholders. Although this study compared learner outcomes using 2 different AHD models, important findings within this study pointed to an understanding of AHD as an inter-subjective learning sphere, orienting learners to each other both socially and academically, and also inspiring medical educators to re-imagine the purpose and scope of the AHD in distributed campus settings.
